# Physician decision-making process about withholding/withdrawing life-sustaining treatments in paediatric patients: a systematic review of qualitative evidence

**DOI:** 10.1186/s12904-022-01003-5

**Published:** 2022-06-24

**Authors:** Yajing Zhong, Alice Cavolo, Veerle Labarque, Chris Gastmans

**Affiliations:** 1 Centre for Biomedical Ethics and Law, Faculty of Medicine, KU Leuven, Kapucijnenvoer 35, block D, box 7001, 3000 Leuven, Belgium; 2 Centre for Molecular and Vascular Biology, Faculty of Medicine, KU Leuven/UZ Leuven, Herestraat 49, 3000 Leuven, Belgium

**Keywords:** Withhold/withdraw life-sustaining treatments, Decision-making, Paediatrics, Palliative care, End-of-life care, Physicians, Perceptions

## Abstract

**Background:**

With paediatric patients, deciding whether to withhold/withdraw life-sustaining treatments (LST) at the end of life is difficult and ethically sensitive. Little is understood about how and why physicians decide on withholding/withdrawing LST at the end of life in paediatric patients. In this study, we aimed to synthesise results from the literature on physicians’ perceptions about decision-making when dealing with withholding/withdrawing life-sustaining treatments in paediatric patients.

**Methods:**

We conducted a systematic review of empirical qualitative studies. Five electronic databases (Pubmed, Cinahl®, Embase®, Scopus®, Web of Science™) were exhaustively searched in order to identify articles published in English from inception through March 17, 2021. Analysis and synthesis were guided by the Qualitative Analysis Guide of Leuven.

**Results:**

Thirty publications met our criteria and were included for analysis. Overall, we found that physicians agreed to involve parents, and to a lesser extent, children in the decision-making process about withholding/withdrawing LST. Our analysis to identify conceptual schemes revealed that physicians divided their decision-making into three stages: (1) early preparation via advance care planning, (2) information giving and receiving, and (3) arriving at the final decision. Physicians considered advocating for the best interests of the child and of the parents as their major focus. We also identified moderating factors of decision-making, such as facilitators and barriers, specifically those related to physicians and parents that influenced physicians’ decision-making.

**Conclusions:**

By focusing on stakeholders, structure of the decision-making process, ethical values, and influencing factors, our analysis showed that physicians generally agreed to share the decision-making with parents and the child, especially for adolescents. Further research is required to better understand how to minimise the negative impact of barriers on the decision-making process (e.g., difficult involvement of children, lack of paediatric palliative care expertise, conflict with parents).

**Supplementary Information:**

The online version contains supplementary material available at 10.1186/s12904-022-01003-5.

## Introduction

Children aged 1–18 years old account for over 30% of the global population [1]. According to the Lancet Commission, almost 2.5 million children die each year from severe illnesses [2]. Given this high child mortality rate and the significant proportion of the world population, it is imperative to establish good end-of-life (EOL) care, in general, and paediatric palliative care (PPC), in particular. Better EOL care can then lead to better quality of life not only for children with life-limiting diseases but also for that of their families [3]. The World Health Organization defined PPC as care directed towards preventing and alleviating the suffering and problems faced by children and their families related to life-threatening conditions [4, 5].

Advances in medicine have markedly increased human survival rates, making it possible now for children with life-threatening conditions to live with a reasonably good quality of life [6, 7]. However, these improvements have also created a care atmosphere in which life-sustaining treatments (LST) can be applied beyond their benefits to patients, possibly leading to prolonged suffering for patients and moral distress for caregivers [8–10]. Making medical decisions at a child’s end of life is a common event in PPC, and for paediatric caregivers, this raises considerable clinical, ethical, sociocultural, legal, and economic issues that challenge medical goals and values [11, 12]. In PPC; an ethically sensitive EOL decision that is frequently made is withholding/withdrawing LST in situations where LST is no longer deemed to be meaningful or effective [13]. Hospital-wide audits of paediatric deaths [14–17] and retrospective studies in paediatric intensive care units (PICUs) [18, 19] revealed that a significant factor associated with children’s deaths is the medical decision to withhold/withdraw LST.

Physicians are regarded as the primary caregivers in paediatric EOL care and they play a pivotal role in deciding whether to withhold/withdraw LST for children. They are responsible for advocating for the best interest of the children under their care [20–23]; providing medical information to stakeholders [20, 24, 25]; and supporting parents and children throughout the care process [20]. Considering the diversity of practices worldwide and the lack of professional consensus regarding LST [21, 26, 27], the decision to withhold/withdraw it is very likely influenced by the personal experiences and attitudes of physicians, who assume clinical and ethical responsibility for their decisions. Hence, understanding physicians’ perspectives when they are faced with the decision to withhold/withdraw LST is key to understanding this ethically sensitive EOL practice. The objective of our study, therefore, is to synthesise qualitative evidence in the literature regarding physicians’ perspectives on the decision-making process for withholding/withdrawing LST in paediatric EOL care.

## Methods

### Design

We performed a systematic review of qualitative studies on physicians’ perspectives towards the decision-making process for withholding/withdrawing LST in paediatric patients. We followed the Peer Review of Electronic Search Strategies (PRESS) guidelines [28].

### Search strategy

**Fig. 1:**
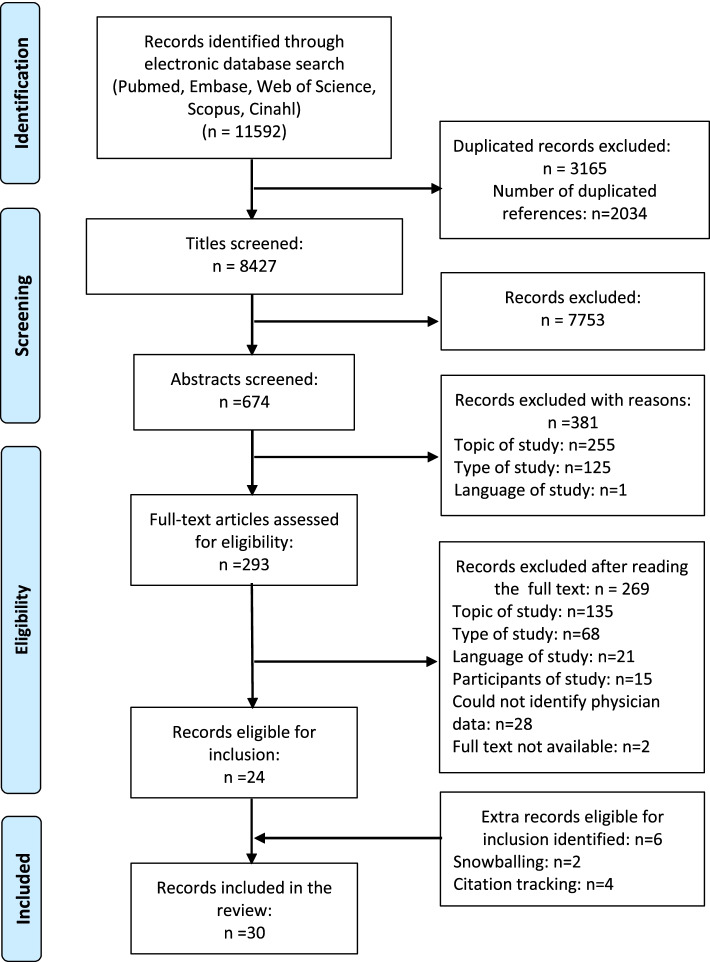
PRISMA flowchart illustrating the process for identifying relevant articles in five electronic databases, and inclusion/exclusion reasons [29]

The first author conducted an extensive search of five electronic databases: Pubmed, Cinahl®, Scopus®, Embase®, and Web of Science™ on March 17, 2021. Before searching, we carried out exploratory manual searches to identify candidate keywords and relevant terms that would guide us in constructing search strings for our target topic. Search strings consisted of Boolean combinations of six categories of search terms: (1) paediatrics; (2) target population (i.e., physicians); (3) end-of-life care; (4) withholding/withdrawing; (5) LST; and (6) perspectives (e.g., perceptions, attitudes, experiences) (Supplementary Material 1). The results of the searches from the separate databases were then merged, and duplicate hits were deleted before conducting title, abstract, and full-text screening. If the full text of a study was not available, we contacted the first author of that study to request a PDF copy of their article. One author replied and attached the full text, two other authors did not reply or attach any documents. The search was complemented with snowballing and citation tracking of reference lists of the included publications to minimise the chance of overlooking relevant publications. The search process followed the overall structure of the preferred reporting items for systematic reviews and meta-analyses (PRISMA) flow diagram (Fig. 1) [29].

### Inclusion and exclusion criteria

Using predefined inclusion and exclusion criteria, two authors (YZ and CG) independently conducted title and abstract screening. On the basis of article titles and abstracts, they agreed on 97 and 92%, respectively, to include or exclude. The first author (YZ) then screened the full text of the candidate articles, came to a provisional decision, and discussed these with the last author (CG) to come to a final decision about inclusion. Disagreements about these full-text decisions were resolved through discussion until consensus was reached. We defined the inclusion and exclusion criteria based on type of studies, participants, and outcome measures (Table 1).

**Table 1:** Inclusion and exclusion criteria for selection of articles on physicians’ perspectives

	Included^**a**^	Excluded
Types of study reported on	• Published empirical studies using qualitative, or mixed-methods designs • Publication language was English • Inclusion was not restricted to a particular time period	• Dissertations, books, book chapters, theoretical articles, guidelines, reviews, case reports, opinion articles, or conference abstracts
Participants in the study	• Practicing physicians in studies sampling their attitudes alone, or • Practicing physicians in studies sampling their attitudes and others including nonphysician clinicians, children, adolescents or parents, only if physicians’ data could be separately extracted	• Studies in which only the perspectives of nonphysician clinicians (e.g., nurses, midwives, trainees, students, children, adolescents, or parents) were sampled
Outcome measures in study reported on	• Physicians’ perspectives, perceptions, attitudes, experiences, preferences, values, feelings, opinions toward the decision-making process about withdrawing/withholding life-sustaining treatments in paediatric patients (children and adolescents: 1–18 years old) • Measures of withdrawing/withholding life-sustaining treatment process in paediatrics and measures focusing on the different steps of withdrawing/withholding life-sustaining treatments in paediatrics separately.	• Measures of only palliative care or end-of-life in paediatrics • Measures of only the complementary alternative medicine or euthanasia in paediatrics • Measures of only withdrawing/withholding life-sustaining treatments in neonates (0 to 1 year old)

^a^ Article screening was not restricted by publication date; the entire possible dates were included in searches of Pubmed, Embase®, Web of Science™, Scopus®, and Cinahl® databases

### Quality appraisal

The quality of included studies was assessed by using the Critical Skills Appraisal Program (CASP) tool [30]. The first author (YZ) independently appraised the articles and discussed uncertain points with the last author (CG) to reach consensus. The methodology of the studies reported on in the included articles was quality appraised and classified as either having high-, moderate-, or low-methodological quality; no studies were excluded on the basis of methodological quality [31].

### Data extraction and synthesis

Three authors (YZ, CG, and AC) performed the data extraction and synthesis process using the updated Qualitative Analysis Guide of Leuven (QUAGOL) approach [32, 33], which normally consists of two coding-process parts conducted over 10 consecutive stages. In the present study, however, we used only the first five stages.

Firstly, we read and re-read the included articles and highlighted relevant information regarding physicians’ views on withholding/withdrawing LST at the end of life; the aim was to generate a holistic understanding of the material. Secondly, we summarised in narrative form the highlighted relevant information with the aim of identifying the main concepts in each publication. Thirdly, we created conceptual schemes for each publication. A conceptual scheme is a synthetic framework of essential elements of the article that answers the research questions [32]. Individual conceptual schemes were interrelated in different ways, producing a web-like structure of the themes overall. Still in this third step, we aimed to ensure that the conceptual schemes were accurate, so we carefully re-read each included article and discussed among ourselves (YZ, CG, and AC) each scheme and fine-tuned it, if necessary. Fourthly, we considered these individual conceptual schemes as a whole to look for inter-relationships, and in turn, to obtain a comprehensive overall answer to our research questions. We purposively focused on our research questions, even if the main topic of some of the included articles was somewhat different from ours. We merged all of the conceptual schemes into a global scheme that integrated the most relevant information about physicians’ perspectives towards the decision-making process for withholding/withdrawing LST in paediatric patients at EOL. In the final stage of the analysis, we synthesised these results into a composite report and prepared it for presentation in the Results section of this review.

## Results

### Study characteristics

Our systematic electronic literature search yielded 30 relevant publications that used a qualitative design in their study (Table 2) [34–63]. Articles reporting on these studies were published between 2004 and 2021 (inclusive). The studies were conducted in 16 countries, representing four continents (North America, Europe, Oceania, and Asia), indicating that considerations on withholding/withdrawing LST in paediatric patients occur on a worldwide scale. Seven studies took place in the USA [39, 40, 43, 49, 55–57]; four each in the UK [41, 45, 47, 53] and Switzerland [34, 36, 50, 52]; three each in France [34, 36, 38] and the Netherlands [44, 51, 63]; two each in Canada [38, 61], Australia [35, 59], Mexico [54, 62], Germany [46, 50], Belgium [34, 36], Luxembourg [34, 36], and Sweden [42, 48]; and one each in Italy [37], Romania [52], Japan [58], and Thailand [60]. Five studies were carried out in more than one country [34, 36, 37, 50, 52].

**Table 2:** Overview of included articles (*n* = 30) on physicians’ perspectives

Study	Location	Aim	Design/Methodology	Participants
Fauriel et al. (2004) [34]	France, Switzerland, Belgium, Luxembourg	To evaluate withdrawal of LST in all nephrology centres in French-speaking European countries	Explorative qualitative approach using semi-directed, face-to-face interviews; Analysis done by two researchers independently	31 paediatric nephrologists involved in LST decisions 17 males; aged 33–61 years, mean 48.5 years; paediatric practice 3–34 years, mean 18.2 years [50 children]
De Graves and Aranda (2005) [35]	Australia	To explore the challenges and complexities when caring for a child with cancer no longer responding to curative therapy, and identify what issues and dilemmas arise when cure is no longer an appropriate goal of treatment	Explorative qualitative approach using participatory group discussions and in-depth interviews; Thematic analysis	6 haematology/oncology consultants [5 registered nurses, 3 haematology/oncology social workers]
Fauriel et al. (2005) [36]	France, Switzerland, Belgium, Luxembourg.	To describe the criteria on which decisions to withhold or withdraw LST were based	Explorative qualitative approach using semi-directed, face-to-face interviews; Analysis done by two researchers independently	46 paediatric nephrologists 30 males, 16 females; aged 33–68 years, mean 50.4 years
Carnevale et al. (2011) [37]	Italy	To describe how LST decisions are made for critically ill children; and how these decisional processes are experienced by physicians, nurses and parents	Explorative qualitative approach using focus group discussions; Follow grounded theory; Thematic analysis	16 physicians practicing in the care of critically ill children 10 males, 6 females; aged 30–57 years, median 42 years; paediatric practice 0.66–28 years, median 13 years [26 nurses, 9 parents]
Carnevale et al. (2012) [38]	France, Canada	To examine how physicians and nurses make decisions about LST for critically ill children and corresponding ethical challenges	Explorative qualitative approach using focus group discussions; Follow grounded theory; Thematic analysis	21 physicians 11 males, 10 females; aged 29–58 years; paediatric practice 0.17–29 years [24 nurses]
Meyer et al. (2012) [39]	United States	To explore how practitioners respond to the question ‘What would you do if this were your child?’	Explorative qualitative approach using descriptive interviews; Thematic analysis	15 physicians [8 nurses, 1 social worker, 1 physical therapist]
Michelson et al. (2013) [40]	United States	To describe issues important in PICU EOL care decision-making and identify possible methods for improving the decision-making process for parents	Retrospective qualitative approach using focus group discussion	13 physicians [23 nurses, 6 social workers, 4 chaplains, 1 child-life specialist, 1 case manager, 18 parents]
Pye (2013) [41]	United Kingdom	To explore perceptions of doctors and nurses working in a pediatric oncology unit regarding experiences and feelings concerning moral distress	Explorative qualitative approach using descriptive phenomenology; Follow Colaizzi descriptive framework; Thematic analysis	4 physicians [4 nurses]
Bartholdson et al. (2015) [42]	Sweden	To describe healthcare professionals’ experiences of ethical issues and ways to deal with these when caring for children with cancer	Explorative qualitative approach using open-ended questions; Qualitative content analysis	15 physicians [72 nurses]
Boss et al. (2015) [43]	United States	To explore pediatric clinicians’ experiences with LST prior to the MOLST mandate and to describe clinician and family concerns and preferences regarding pediatric MOLST	Explorative qualitative approach using focus group discussions; Analysis done by two researchers independently	69 physicians [27 nurses]h8
De Vos et al. (2015) [44]	The Netherlands	To answer the questions: How do physicians and parents communicate about decisions to withhold or withdraw LST, and to what extent do parents share in the decision-making process?	Prospective explorative approach; Qualitative and quantitative analysis	27 physicians 15 males, 12 females; paediatric practice, 12 were 0–5 years, 2 were 5–10 years, 13 were ≥ 10 years [37 parents]
Forbat et al. (2015) [45]	United Kingdom	To explore health professionals and family experiences of conflict in pediatric services	Explorative qualitative approach using semi-structured interviews; Thematic analysis	20 physicians [10 nurses, 8 parents, 3 chaplains, 2 lawyers, 2 Patient Advice and Liaison Service, 1 hospice head of care]
Lotz et al. (2015) [46]	Germany	To investigate the attitudes, barriers, and benefits as well as requirements for pediatric ACP	Explorative qualitative approach using semi-structured interviews; Content analysis	9 physicians [6 nurses, 2 social professionals]
Mitchell & Dale (2015) [47]	United Kingdom	To investigate current practice in ACP and how this might be improved, by exploring the experiences and perceptions of senior PICU medical and nursing staff who are frequently involved in managing EOL care for children and young people	Explorative qualitative approach using semi-structured interviews; Thematic content analysis	8 PICU consultants [6 nurses]
Bartholdson et al. (2016) [48]	Sweden	To explore healthcare staff’s experiences of participating in ECR sessions in childhood cancer care	Explorative qualitative observations of ethics care reflection sessions and qualitative interviews; Follow grounded theory	15 physicians in ECR session, 2 physicians in individual interviews 13 males, 2 female in ECR; 2 males in interviews [18 nurses, 1 therapist, 1 psychologist in ECR session, 8 nurses in individual interviews]
Bateman et al. (2016) [49]	United States	To describe communication between physician and the actor parent of a standardized 8-year-old patient in respiratory distress who is nearing the EOL	Explorative qualitative approach using high-fidelity simulation; Follow grounded theory; Thematic analysis	13 paediatric emergency medicine and paediatric critical care fellows 8 males, 5 females
Lotz et al. (2016) [50]	Germany, Switzerland	To investigate which factors paediatricians apply in deciding about medical indication, and how they manage conflicts with parents	Explorative qualitative approach using low-structured and case-based focus group discussions; Content analysis	17 experienced paediatricians 12 males, 5 females; mean age 44 years, mean practice experience 16 years
Zaal-Schuller et al. (2016) [51]	The Netherlands	To compare the experiences of parents and physicians who were involved in the EOL decision-making process of the same child with profound intellectual and multiple disabilities	Retrospective qualitative approach using semi-structured interviews; Coding done by two researchers independently	11 physicians 2 males, 9 females; aged 40–60 [14 children, 17 parents]
Badarau et al. (2017) [52]	Switzerland, Romania	To explore views of parents and physicians on decision-making in paediatric oncology	Explorative qualitative approach using semi-structured interviews; Thematic analysis	26 physicians Aged 30–62 [37 parents]
Birchley et al. (2017) [53]	United Kingdom	To critically describe the way in which the best interests standard operates in PICU	Explorative qualitative approach using interviews; Thematic analysis	10 physicians [14 parents, 8 nurses, 7 clinical ethics committee]
Cicero-Oneto et al. (2017) [54]	Mexico	To explore in-depth the EOL decision-making process and to identify the ethical principles that guide decision-making	Explorative qualitative approach using semi-structured, in-depth interviews; Thematic analysis	13 oncologists 5 males; aged 32–52; practical experience 1–20 years, median 7 years [13 parents, 6 adolescents]
Odeniyi et al. (2017) [55]	United States	To identify oncologists’ and intensivists’ perceived barriers to, and resources for, communication with families of children with cancer, and how the oncologist-intensivist relationship impacts communication and initiation of goals of care discussions	Explorative qualitative approach using semi-structured interviews; Thematic analysis	10 paediatric oncology and intensive care physicians 5 males
Richards et al. (2018) [56]	United States	To understand how critical care physicians balance and integrate the interests of the child and family in decisions about LST	Explorative qualitative approach using semi-structured interviews; Thematic and content analysis	22 physicians 15 males, 7 females; work experience 10 have ≤5 years, 12 have ≥5 years
Needle et al. (2019) [57]	United States	To explore the perspectives of PICU and hematopoietic stem cell transplant healthcare professionals as related to informed decision making of adolescents and the value of advance directives in paediatric practice	Explorative qualitative approach using focus group discussions; Content analysis	15 physicians 8 males; aged 29–62 years, mean 40 years; practice experience 1–31 years, mean 10 years
Sasazuki et al. (2019) [58]	Japan	To delineate the decision-making processes that paediatricians apply when treating children with life-threatening conditions and the psychosocial experience of paediatricians involved in such care	Explorative qualitative approach using semi-structured interviews; Comprehensive qualitative and content analysis	15 paediatricians 14 males, 1 female; aged 30–54 years
Ekberg et al. (2020) [59]	Australia	To explore how discussions about deterioration are managed within actual paediatric palliative care consultations.	Explorative observative approach; Follow grounded theory; Conversation analysis	7 healthcare professionals
Jongaramraung et al. (2020) [60]	Thailand	To investigate EOL decisions for children in PICU	Explorative qualitative approach using in-depth interviews; Follow naturalistic inquiry	2 physicians [17 nurses]
Orkin et al. (2020) [61]	Canada	To develop an in-depth understanding of the ACP experience for children with medical complexity	Explorative qualitative approach using semi-structured interviews; Content analysis	8 physicians [14 mothers, 2 nurses, 1 social worker]
Fay et al. (2021) [62]	Mexico	To analyse the ways in which pediatric patients have agency in relation to their parents and palliative care professionals within the hospital setting, as well as the structural circumstances that constrain said agency	Explorative qualitative approach using semi-structured interviews; Follow hospital ethnography; Thematic analysis	1 general practitioner and 1 paediatrician Pediatric experience, general practitioner 10 months, paediatrician 5 years [2 psychologists]
Verberne et al. (2021) [63]	The Netherlands	To explore how parents and healthcare professionals currently anticipate the future of the child and family in paediatric palliative care	Explorative qualitative approach using semi-structured interviews; Thematic analysis	20 physicians [42 parents, 13 nurses, 1 psychologist, 1 child-life specialist]

*LST* Life-sustaining treatment, *PICU* Paediatric intensive care unit, *EOL* End-of-life, *ACP* Advance care planning, *ECR* Ethics care reflection, *MOLST* Medical orders for life-sustaining treatment

Relevant data were collected through different qualitative methods. Most studies used semi-structured interviews to sample physicians’ perceptions. Other studies used focus group discussions, open-ended questions, and conversations.

The analysis approach was reported in 23 studies [35, 37–39, 41, 42, 44–47, 49, 50, 52–59, 61–63]. Thirteen studies used thematic analysis [35, 37–39, 41, 45, 49, 52–55, 62, 63]. Six studies used content analysis to analyse qualitative data [42, 46, 50, 57, 58, 61]. Two studies used thematic analysis as well as content analysis [47, 56].

As a whole, 499 physicians participated in the studies reported on in the included articles. Of these 499 physicians, 116 were paediatric intensivists, 77 were paediatric nephrologists, 71 were paediatric haematologists or oncologists, 21 were general paediatricians, 17 were neonatologists, 13 were paediatric neurologists, 6 were paediatric cardiologists, 6 were general practitioners, 1 was a paediatric chronic disease specialist, 1 was a paediatric rehabilitation specialist, and 1 was a paediatric revalidation specialist. The practicing specialty of the remaining 169 physicians was not stated. The study sample size ranged from 2 to 69. Fourteen of the articles reported the gender of the physicians [34, 36–38, 44, 48–51, 54–58]. With the exception of two studies [51, 54], most of them had more male physician subjects.

Seven studies included only physicians [36, 49, 50, 55–58]; the remainder of the studies included other healthcare professionals, parents, or additionally children. Of the 30 included articles, five reported the physicians’ attitudes regarding withholding/withdrawing LST [39, 46, 56, 57, 63]. The remaining articles reported physicians’ experiences about withholding/withdrawing LST.

### Methodological quality

Table 3 summarises the results on quality assessment of all included publications. Twenty-two studies were deemed high quality, and eight were evaluated as moderate quality. All included studies were carried out using appropriate methodologies. With a few exceptions, the majority of studies contained a clear statement of aims and findings; most of the studies obtained ethical approval from ethics committees and informed consent from participants. Most of the studies developed significant recruitment strategies and rigorously analysed their data. However, in 23 studies, researchers considered their own roles, potential biases, reactions to events and implications of changes in the study design inadequately.

**Table 3:** Quality assessment of included articles using CASP^a^

Author	1. Was there a clear statement of the aims of the research?	2. Is a qualitative methodology appropriate?	3. Was the research design appropriate to address the aims of the research?	4. Was the recruitment strategy appropriate to the aims of the research?	5. Was the data collected in a way that addressed the research issue?	6. Has the relationship between researcher and participants been adequately considered?	7. Have ethical issues been taken into consideration?	8. Was the data analysis sufficiently rigorous?	9. Is there a clear statement of findings?	10. How valuable is the research?	Overall assessment
1. Fauriel et al. (2004) [34]	Y^b^	Y	U	Y	Y	N	Y	Y	Y	Y	27^b^ H
2. De Graves & Aranda (2005) [35]	Y	Y	Y	Y	Y	Y	Y	U	Y	Y	29 H
3. Fauriel et al. (2005) [36]	Y	Y	U	U	Y	N	Y	Y	Y	U	25 M
4. Carnevale et al. (2011) [37]	Y	Y	Y	Y	Y	Y	Y	Y	Y	Y	30 H
5. Carnevale et al. (2012) [38]	Y	Y	Y	Y	Y	Y	Y	Y	Y	Y	30 H
6. Meyer et al. (2012) [39]	Y	Y	U	Y	Y	N	Y	Y	Y	Y	27 H
7. Michelson et al. (2013) [40]	Y	Y	U	Y	Y	N	U	Y	U	Y	25 M
8. Pye (2013) [41]	Y	Y	Y	U	Y	N	N	U	U	Y	23 M
9. Bartholdson et al. (2015) [42]	Y	Y	U	Y	Y	N	N	Y	Y	Y	25 M
10. Boss et al. (2015) [43]	Y	Y	Y	U	Y	Y	N	Y	Y	U	26 H
11. De Vos et al. (2015) [44]	Y	Y	Y	U	U	N	Y	Y	Y	Y	26 H
12. Forbat et al. (2015) [45]	Y	Y	U	U	Y	N	Y	Y	Y	Y	26 H
13. Lotz et al. (2015) [46]	Y	Y	U	Y	Y	N	Y	U	Y	U	25 M
14. Mitchell & Dale (2015) [47]	Y	Y	Y	N	Y	Y	U	Y	Y	Y	27 H
15. Bartholdson et al. (2016) [48]	Y	Y	Y	U	Y	U	Y	U	Y	Y	27 H
16. Bateman et al. (2016) [49]	Y	Y	Y	Y	Y	N	Y	Y	Y	U	27 H
17. Lotz et al. (2016) [50]	Y	Y	Y	Y	Y	U	U	U	Y	Y	27 H
18. Zaal-Schuller et al. (2016) [51]	Y	Y	U	Y	Y	N	U	Y	Y	Y	26 H
19. Badarau et al. (2017) [52]	U	Y	Y	Y	Y	N	Y	Y	Y	Y	27 H
20. Birchley et al. (2017) [53]	Y	Y	Y	Y	Y	N	Y	U	Y	Y	27 H
21. Cicero-Oneto et al. (2017) [54]	Y	Y	Y	Y	Y	N	Y	Y	Y	Y	28 H
22. Odeniyi et al. (2017) [55]	Y	Y	U	N	U	N	U	Y	Y	Y	23 M
23. Richards et al. (2018) [56]	Y	Y	U	U	U	N	Y	Y	Y	U	24 M
24. Needle et al. (2019) [57]	U	Y	Y	U	Y	Y	Y	Y	Y	Y	28 H
25. Sasazuki et al. (2019) [58]	Y	Y	Y	Y	Y	N	U	Y	Y	U	26 H
26. Ekberg et al. (2020) [59]	Y	Y	Y	Y	U	Y	Y	U	U	Y	27 H
27. Jongaramraung et al. (2020) [60]	Y	Y	Y	Y	U	N	Y	U	Y	Y	26 H
28. Orkin et al. (2020) [61]	Y	Y	Y	U	Y	N	Y	Y	Y	Y	27 H
29. Fay et al. (2021) [62]	Y	Y	Y	U	U	N	Y	U	Y	U	24 M
30. Verberne et al. (2021) [63]	Y	Y	Y	U	U	N	Y	Y	Y	Y	26 H

*Y* Yes, *U* Unclear, *N* No, *H* High, *M* Moderate

^a^Critical Appraisal Skills Program (CASP UK, 2013); scoring: yes = 3; unclear = 2; no = 1

^b^Total score

### Main findings

Our QUAGOL-guided analysis identified four components of physicians’ perceptions on the decision-making process about withholding/withdrawing LST in paediatric patients: stakeholders, structure, ethical values, and influencing factors (Table 4). We used these four components to organise the presentation of our findings.

**Table 4:** Components of physicians’ perceptions^a^ identified in the QUAGOL-guided analysis

Components	Included publications
**Who should be involved in the decision-making process, what are their roles, and how do they experience their involvement**	
**Physicians**	[34, 36–44, 46–48, 50–55, 57–63]
**Parents**	[34–40, 42–44, 47, 50–56, 60–63]
**Child**	[34, 36–38, 46, 47, 50, 52–54, 57, 61, 62]
**Structure of the decision-making process about withholding/withdrawing LST**	
**Early preparation for the decision-making via ACP**	[46, 47, 57, 61]
Facilitators	[57, 61]
Barriers	[46, 47, 57, 61]
**Decision-making process with information delivery and receipt**	[34, 36, 38, 39, 43, 44, 48, 49, 51, 53, 54, 56, 59–61, 63]
Understanding the medical situation of the child	[34, 36, 39, 43, 44, 48, 51, 54, 59, 60, 63]
Manner of exchanging and discussing relevant information	[34, 38, 49, 53, 54, 56, 60, 61]
**Making final decisions**	[36–38, 44, 52, 54, 56, 60, 63]
Withholding/withdrawing LST	[36, 37, 44, 54, 56, 60, 63]
Continuing LST	[36–38, 44, 52, 56, 60]
**Ethical values that are balanced in the decision-making process**	
**Best interests of the child**	[34, 36, 37, 42, 44, 46, 47, 50, 51, 53–56, 58, 60]
**Best interests of parents**	[34–40, 42, 47, 50–56, 59–63]
**Factors influencing decision-making**	
**Facilitators**	[34, 36–44, 47, 50–57, 59–63]
**Barriers**	[34, 35, 37–39, 42, 43, 45–47, 49–55, 58–63]

^a^ Physicians’ perceptions on the decision-making process about withholding/withdrawing LST in paediatric patients

### Who should be involved in the decision-making process, what are their roles, and how do they experience their involvement

Most of the included publications reported the physicians’ perceptions on who they felt the key stakeholders are that should participate in the decision-making, what the nature of their roles are, and how these stakeholders experience their participation in the decision-making process. We found that almost all physicians held the viewpoint that decisions about withholding/withdrawing LST should be made jointly by physicians, parents, and patients (given that the latter are old enough to participate meaningfully). How the physicians defined ‘shared decision-making’ varied, depending on a given stakeholder’s specific role and level of involvement in the decision-making process.

#### Physicians

A large number of physicians stated that they played a major role in the decision-making [34, 37, 38, 40–44, 48, 51, 52, 54, 59, 60, 62, 63]. In three publications, most of the physicians preferred the decision-making to be shared with colleagues; that is, via medical team collaborations [34, 38, 52]. In the early stages of the decision-making, physicians described their primary roles as the one diagnosing diseases, initiating and promoting open discussions with parents about their child’s condition (especially if they foresaw a potential deterioration), facilitating the development of a future care plan for the child, and allocating care resources [38, 39, 43, 44, 48, 52, 54, 59, 60, 63]. Later on in the decision-making, physicians felt medically responsible for making the final decision on withholding/withdrawing LST [34, 37, 38, 40–42, 44, 51, 52, 54, 60, 62]. While most physicians preferred sharing their final decisions with parents, and even with patients if appropriate [34, 38–40, 42, 47, 50–54, 57, 60–63], some of them viewed decisions about futile LSTs as strictly medical and preferred not sharing their decisions of withholding/withdrawing LST with parents [44, 51, 52, 54].

Physicians also reported playing other roles. For example, they oriented and reassured parents during the decision-making process [34, 36, 38–40, 42–44, 48, 51, 52, 54, 55, 60, 63]. Physicians provided parents with appropriate information about their child’s critical condition to assist them in making decisions that are beneficial for the child [34, 36, 39, 40, 42–44, 48, 52, 54, 55, 60, 63]. Physicians empathised with parents, reassuring them that their decisions were correct in order to ease the parents’ feelings of guilt [34, 36, 38, 40, 44, 55, 63].

Physicians experienced both positive and negative feelings when being involved in the decision-making process about withholding/withdrawing LST [36–39, 41, 46, 47, 50, 57, 58, 61]. Some physicians repeatedly characterised their experiences as being on a ‘mission’ to care for severely ill children [44, 58]. Other physicians reported feeling more confident, because they had acquired more clinical experience [41] or feeling more secure when a clear legislative framework for decision-making was in place [36, 37, 50].

Nevertheless, many more physicians expressed negative feelings, including struggling with carrying the weight of responsibility for making decisions to withhold/withdraw LST [36, 37, 41] and fearing therapeutic obstinacy when they knew a given treatment would not benefit the patient [37, 41, 44, 46, 57]. Physicians also reported being fearful of making errors [37–39, 46, 57], especially when they were unfamiliar with the child as a patient [46]. They were also frightened when they were unfamiliar with the child’s best interests but were obliged to make decisions with little time available [58]. Some physicians reported feeling isolated and lonely when making these complex and delicate decisions [37, 41].

#### Parents

Overall, physicians agreed that parents should participate in the decision-making on withholding/withdrawing LST [34, 36, 38–40, 42, 47, 50–56, 60–63]. They felt that these decisions are very personal for parents and that they should reflect family values [39]. Therefore, parents were given opportunities to discuss their values and preferences with members of the healthcare team [34, 36, 38–40, 42, 47, 50–56, 60–63]. Especially in cases of poor prognoses or unavailable standard treatments, physicians felt that the parents and patients should play a prominent role in the decision-making [51, 52, 61]. The main reason was that physicians knew that giving parents a key role was in the best interest of the child [47, 51, 55, 56]. As parents know their child the best, they could more accurately interpret and communicate their child’s behaviours, judge the degree of their child’s suffering, and express concerns based on their observations [47, 51, 56].

Some physicians reported that some parents authorised them to make decisions about withholding/withdrawing LST in order to avoid being ultimately responsible for these decisions [34, 37, 38, 40, 50, 52, 54, 63]. In general, physicians struggled with placing the burden of major EOL decisions on parents, because most parents do not have sufficient medical knowledge to fully understand all the implications of their decisions, and they would have to live with the consequences of their decisions [34, 40, 53, 55, 61, 63].

De Graves & Aranda reported that communicating with parents is considered to be the biggest challenge in paediatric oncology [35]. Our present analyses corroborated those findings. Physicians were concerned about when and how to discuss EOL decisions with parents and how to provide them with support [51, 53, 55]. As parents may still hope their child can be cured, they may not be ready to accept palliative care [35]. In the study of Boss et al. [43], for instance, physicians felt that families and the physician-parent relationship might become strained or damaged by discussing withholding/withdrawing LST with parents if their child has a less serious or mild condition.

Some physicians also described what parents experienced during the decision-making process about withholding/withdrawing LST. Physicians said parents experienced positive, negative, or mixed feelings when involved in the decision-making process. In de Vos et al. [44], physicians reported that most parents understood and were not surprised about the inevitable need to withhold/withdraw LST. However, in other studies, physicians stated that parents faced difficult situations. For instance, being confronted with therapeutic futility could make parents psychologically vulnerable and could hinder them from participating in the decision-making [39, 54]. Physicians perceived that for many families the decision-making process was a ‘roller-coaster ride’ of emotional ups and downs, interspersed with despair and grief as the possibility of death became a distinct reality [35].

#### Child

Only eight publications discussed what physicians did or could do to involve paediatric patients in the decision-making process, such as informing them about their critical condition and considering their opinion about future care plans [34, 47, 50, 52, 54, 57, 61, 62]. Seven of these publications targeted at adolescents [34, 47, 50, 52, 54, 57, 62]. Because of their older age (compared to a child’s), respect for autonomy was regarded as more important for adolescent patients [50, 57, 62]. However, some physicians were hesitant to involve paediatric patients in the decision-making process [36, 37, 46, 50, 52–54, 57, 62], because they were concerned about medicolegal liabilities and the adolescent’s capacity to make decisions [36, 37, 46, 50, 53, 54, 57]. For instance, in Mexico the law clearly states that parents are legally responsible for making decisions about withholding/withdrawing LST for their child, but it does not specify the age at which minors should be involved in the decision-making process [54].

Determining when and how children should participate in treatment decisions was described as a real challenge for physicians [36–38, 46, 50, 52, 54, 57, 62]. In the study of Fay et al. [62], physicians reported that patients were afraid to ask questions about their condition, because their parents did not tell them anything about their condition. Hence, parents revealed only what they wanted to reveal to their child rather than revealing what their child actually wanted to know [62]. In this context, parents assessed their child’s capacity to make decisions [62]. Understandably, physicians were sceptical of their child’s autonomy, as they doubted whether a minor could really understand and process the necessary information needed to make a well-informed decision [57]. The great variability in different minor patients’ capacity for making informed decisions only increased the complexity of deciding whether to include paediatric patients in the decision-making process [57].

### Structure of the decision-making process about withholding/withdrawing LST

Our analyses showed that from the physicians’ perspective, the decision-making process could be divided into three stages: (1) early preparation via advance care planning (ACP); (2) information giving and receiving (this is the main decision-making process where stakeholders exchange and discuss information); and (3) arriving at the final decision about withholding/withdrawing LST. These will be described in more detail next.

#### Early preparation for the decision-making via ACP

Physicians identified major aims of the ACP process. The first aim of ACP was to discuss values, preferences, and goals of care with parents and the child through an exchange of relevant information [57, 61]. In this way, physicians were well-informed about what treatments the parents and the child would find appropriate at the end of life [57]. The second aim of ACP was to improve the quality of care and to make decisions that were in the best interests of the child; that is, by avoiding unnecessary suffering resulting from emergency or intensive care interventions [46, 47]. Physicians identified both facilitators and barriers in realising these aims.

##### Facilitators

Physicians mentioned four key facilitators in the early preparation stage of decision-making via ACP: (1) shared decision-making between healthcare professionals (HCPs) and parents [61]; (2) supportive setting (i.e., comfortable location, and sufficient time and opportunities for all team and family members to meet) [61]; (3) early, ongoing conversations starting at the time of diagnosis and when patients were still well enough to discuss their condition, values, preferences, and goals [57, 61]; (4) involvement of HCPs with special training and sufficient expertise in ACP discussions [57, 61].

##### Barriers

Physicians perceived three barriers that could hinder the early preparation stage of decision-making via ACP. The first key barrier related to uncertainty issues regarding the early recognition of the life-threatening condition of the child. Physicians were regularly confronted with a lack of diagnostic precision and with insufficient time to evaluate the child’s illness trajectory as precisely as they desired [61]. Consequently, ACP conversations were started only when patients were already seriously ill [46, 47, 57].

The second barrier relates to difficulties HCPs experience in reaching consensus about appropriate care goals [47, 57]. Different clinical judgements about clinical risk management and the effectiveness of medical interventions and different ethical attitudes among HCPs represented a barrier in their desire to reach a consensus in the healthcare team [47].

The third barrier involved four factors that physicians perceived to hinder HCPs and parents from reaching consensus about care goals [47, 57]: (1) physicians’ not fully knowing the care goals of parents and patients [57]; (2) parents’ varying and vacillating perspectives towards EOL decisions [57]; (3) parents’ unrealistic expectations about PICU admissions [47]; and (4) parents’ cultural, religious, and social background [57].

#### Decision-making process with information delivery and receipt

When physicians diagnosed that a child in their care was approaching the EOL, they initiated EOL discussions alone with the parents or in conjunction with the paramedical team. Shared understanding of the medical situation of the child and the way relevant information is exchanged and discussed were identified as elements that contributed to the quality of the decision-making.

##### Understanding the medical situation of the child

We identified four essential steps that physicians considered to be important to promote a shared understanding of the child’s medical situation. First, during meetings with families, physicians initially discussed what the parents understood about the child’s illness and the progression of their condition. They did this to assess whether parents could accept the eventuality of their child’s prognosis [60].

Second, physicians provided a medical update of the child’s current condition and prognosis based on their clinical observations of the child’s discomfort, ability to communicate, and responses to advanced medical treatment [39, 43, 44, 48, 60, 63]. Asking and answering questions about the efficacy and futility of possible interventions enabled physicians to enhance their understanding of the clinical and caring situation of the child (e.g., experience of pain, quality of life) and that of the family’s (e.g., psychosocial situation, ethical concerns) [48]. This exchanging of information also better prepared parents for possible future decisions that they might face about withholding/withdrawing LST [34, 36, 39, 44, 54, 60, 63].

Third, physicians considered it to be important to promote a shared understanding of the child’s medical situation related to disease progression. Physicians perceived acute deterioration as the most common reason to initiate the discussion about withholding/withdrawing LST. Physicians created opportunities for parents to discuss their child’s deterioration, as these discussions might facilitate future care planning [51, 59].

Fourth, before final decisions could be made, physicians thought it was important first to provide parents with an update on possible treatment options (e.g., maximum therapeutic treatments, withholding/withdrawing LST) and to lay out the consequences of each treatment option for the child (e.g., negative effects of paediatric intensive care admissions) [43, 48, 60, 63]. In the study of de Vos et al. [44], most physicians asked parents at the end of a family meeting whether they had additional questions.

##### Manner of exchanging and discussing relevant information

Besides the content of the information being exchanged, physicians emphasised that it was important to be aware of *how* to exchange and discuss relevant information on withholding/withdrawing LST during the different stages of the decision-making process. Physicians emphasised that information needed to be conveyed in a simple and concise way to help parents understand the medical perspective [34, 53, 60, 61]. Therefore, they suggested using clear and unambiguous language without medical jargon and to use visual aids, such as medical imaging scans [34, 60, 61].

Physicians also stated that it was important not to withhold/withdraw information about the child’s condition; rather, it was important to paint an accurate, complete picture of the child’s condition and to do so repeatedly and in a consistent way [49, 54, 60, 61]. Finally, physicians reported that communication with parents should be adapted to the evolving situation of the child and the parents’ state of mind [38, 53, 54, 56]. For instance, if the parents disagreed with the physician’s views on the best interests of the child, physicians said they should try to reframe the care plan in terms they thought would be more acceptable to parents [53]. However, those authors also said that physicians should also be more direct about withholding/withdrawing LST [56]. Moreover, when a patient’s condition was severe, they said that the delivery of information should be unvarnished and blunt [56].

#### Making final decisions

When it came to the stage of making a final decision about whether to withhold/withdraw LST at the end of life of a paediatric patient, physicians considered two major options.

##### Withholding/withdrawing LST

When medical treatments became clearly futile at EOL, the majority of physicians supported a final decision concerning withholding/withdrawing LST to limit the child’s suffering [37, 44, 63] and to improve the child’s living conditions [36, 37, 54]. To realise this improvement, physicians said that care goals should be reset [56]. They acknowledged that the final decision to withhold/withdraw LST can be extremely difficult for parents. However, parents who initially argued for extraordinary treatments to be tried, eventually agreed that there were no longer reasonable options to consider when their child failed to respond [37, 44, 60]. Some parents preferred to withhold/withdraw LST and take their child back home so EOL care and death could happen there [54, 60] (sometimes expressed as keeping their child’s soul at home [60]).

Richards et al. [56] described three strategies used by physicians to help parents deal with these difficult decisions. First, they limited the range of options that they thought were futile, because they did not want to give the parents false hope. Second, when parents felt that they could not make decisions about withholding/withdrawing LST because of religious reasons (even if they agreed with the physician’s decision), physicians helped them make it. Third, physicians delayed making a final decision, because some parents needed to witness their child’s dying to understand that death was inevitable.

##### Continuing LST

Our analysis revealed that a minority of physicians supported the decision to continue LST, even in acute situations or when they were in doubt of the child’s condition. Opting to continue LST was the standard protocol to ensure that patients had been afforded all possible opportunities to be cured [37, 38, 52, 56]. A small number of physicians argued that it was important to continue LST, because they felt they possessed no right to decide whether any person should live or die [36, 44]. According to physicians in the study of Jongaramraung et al. [60], some parents wanted extraordinary therapeutic treatment to be tried, because they were not ready to accept the likely fact that their child was dying.

### Ethical values that are balanced in the decision-making process

Physicians reported that they felt a need to balance their ethical values, including advocating for the best interests of the child and those of the parents. Within this aim, they articulated their values and commitments as professionals to make ethically sound decisions.

#### Best interests of the child

Physicians emphasised that advocating for the best interest of the child was the main driver of their decision-making when considering LST at their paediatric patients’ EOL [47, 53–55]. Specifically, our analysis revealed that physicians recalled their experiences of handling similar cases in the past and compared them against published cases. This kind of self-evaluation helped them reach a decision by balancing the expected benefits and burdens of LST [36, 50, 56]. They perceived specific factors related to the patients’ clinical condition as being essential during the decision-making process: (1) laboratory results and radiological investigations of the child [36]; (2) medical history, diagnosis, and acute deterioration of the child [50, 51]; (3) the child’s response to already-attempted treatments and the possible reversibility of current symptoms [36, 50, 60]; and (4) the risks of complications from new treatments being considered [50]. Physicians reported trying to avoid prolonging the child’s suffering; and by carefully observing the child’s comfort, they aimed to improve their current and future quality of life [36, 44, 54, 56, 58]. They did this by considering their wishes and desire to continue to live [34, 50]. Physicians adopted an advocacy role when presenting to parents clinical perspectives related to the best interests of the child [53].

Physicians also mentioned that it was not always easy to advocate for the best interests of the child [37, 42, 58]. Several obstacles in this regard that physicians faced were difficulties in imagining the pain of a child’s suffering from particular diseases (e.g., neuromuscular disease) [58]; hesitations about what was best for the patients in a particular situation or in a need to comply with legal regulations [37, 46, 50, 53].

#### Best interests of parents

Physicians also stressed the importance of advocating for the best interests of the parents [47, 50, 53, 55, 56]. Therefore, parents’ attitudes towards withholding/withdrawing LST, as well as their well-being, were considered in the decision-making process [34, 36, 38–40, 42, 50–54, 59–63]. Physicians reported that parents needed some time to accept their child’s condition before making a final decision [56]. They needed to gain a sense of control over the decisions made to reach a sense of peace about their decision [56]. Therefore, some physicians tried to instil hope in the parents, because hope that their child would survive often exists—despite disease progression—even up to moments just before death [35]. They considered how the consequences of their decisions would affect parents, trying to avoid placing more burden of responsibility on the parents, or leading them towards a direction that would bring future regrets [34, 37, 38, 40, 50, 55, 63].

### Factors influencing decision-making

Physicians described some factors related to patients, parents, and physicians that facilitate or impede the decision-making process about withholding/withdrawing LST at their paediatric patients’ EOL. We refer to these as facilitators and barriers.

#### Facilitators

Physicians stated that a good collaborative relationship with the medical team and parents was essential in helping them make decisions about withholding/withdrawing LST at EOL.

For parents, physicians mentioned three facilitators. One facilitator was routinising LST discussions and sharing their decisions with parents. Physicians perceived this as being very helpful for maintaining a long-lasting trusting relationship with them [39, 43, 51]. Although disagreements often arose during discussions with parents, physicians emphasised not all disagreements were concerning. Some disagreements stimulated physicians to think about more suitable alternatives for the child’s specific situation [51]. A second facilitator was providing practical and psychosocial support to parents by encouraging them to spend more time with their child and to take advantage of psychosocial resources, including other families and potentially helpful persons (e.g., a priest) [39]. A third facilitator for the decision-making process was parents’ experience with and understanding of (embodied knowledge) their child’s previous treatments [51, 54, 57].

The deterioration in the child’s physical appearance was one of the most frequently identified child-related facilitators, according to physicians. This factor enabled parents to witness their child’s deterioration and facilitated their comprehension of their child’s medical condition [53, 54]. This acceptance by the parents validated the physicians’ clinical decision to withhold/withdraw LST, with the greater aim of promoting the child’s best interests [47, 53–55].

#### Barriers

Physicians also reported barriers that hindered their decision-making about withholding/withdrawing LST at EOL in their paediatric patients. First, lack of training in palliative care and appropriate EOL communication was the most frequently identified barrier for physicians’ decision-making [38, 42, 43, 46, 47, 52, 54, 55, 62]. Physicians reported misunderstanding when to start palliative care. For example, some thought palliative care starts only when the child is dying, while others misunderstood the legal and ethical specifics of do-not-resuscitate orders [43, 62]. Thus, they had difficulties in supporting their close HCPs or those of other units, because they themselves were ambivalent about palliative care [42, 62]. Further complicating the implementation of palliative care in children were the limited use of guidelines for making ethically appropriate decisions [58] and inadequately developed care models for children with life-threatening diseases [37].

A second barrier, as described by physicians in the Zaal-Schuller et al. study [51], was conflicts between physicians and parents, which significantly hindered decision-making. For instance, sometimes parents forbade a LST, even though physicians still anticipated that the child’s condition still had a realistic chance of improving. In other cases, parents wished every possible LST to be attempted, even though physicians considered all treatments to be futile.

Conflicts between physicians and parents can have different roots. The most frequently identified source for disagreements was that many parents lacked sufficient medical background knowledge to clearly evaluate the physician’s proposed course of action/inaction [35, 49, 51, 54]. Awkward, confusing, and misleading discussions with physicians [49]; clinical uncertainties regarding prognosis; and unforeseeable complications contributed to these misunderstandings [35, 51]. Another factor that complicated decision-making was differences in ethnic, religious, and/or linguistic backgrounds of physicians and parents [42, 51].

Physicians in the study of Forbat et al. [45] described three levels of conflicts between physicians and parents: mild, moderate, and severe conflicts. Mild conflicts contributed to the physician’s poor management of their relationship with families. Previous conflicts about treatment decisions caused physicians and parents to be more conscious of the potential risk of escalating disagreements. Moderate conflicts deteriorated the trust between physicians and parents. These kinds of conflicts arose because parents questioned and disagreed with the treatments proposed by physicians, leading to frequently revisited arguments. The arguments would become unshakeable when physicians and parents continued to defend their position forcefully and were unwilling even to listen or compromise. Severe conflicts disintegrated working relationships, because both physicians and parents shifted their focus from the child’s best interest to the conflict itself. Ad hominem (e.g., physical attack) and professional (e.g., reporting to the press or professional regulators) threats were carried out.

Physicians mentioned three options they used when dealing with parental dissent about withholding/withdrawing LST. First, they tried to comply with the parents’ request. Most physicians chose this option when parents decided to refuse the indicated LST [34, 38, 39, 42, 50–54, 59–63]. They accepted parents’ rights to reject physicians’ clinical recommendations and agreed with their superior ability to assess the child’s presumed wishes [50]. Physicians were motivated to comply with parents’ requests to use non-indicated LST in order to maintain trust and to build a close relationship with parents; they wanted to discourage ‘doctor shopping’ because of the known harm that would cause to the child and to try to avoid legal problems [50, 53].

The second option for dealing with parental dissent about withholding/withdrawing LST was to re-focus on the child’s best interests and to seek a consensus decision with the parents. For example, this option was manifested by delaying to make final decisions, continuing negotiations with parents, and sharing decision-making [50, 53]. Physicians felt that resolving conflicts about a child’s best interest in the absence of court involvement really represented personal and institutional success [53].

The final option at the disposal of physicians was to override the parental decision, because physicians have the ethical right to refuse to act against their own conscience, their medical expertise, and their duty to deliver competent care and to avoid harm to the child [50].

## Discussion

The results of our systematic review rest on an extensive analysis of 30 qualitative, predominately high-quality, publications appearing between 2004 to 2021. The QUAGOL-guided [32, 33] analysis of these 30 articles form the basis of our comprehensive description of physicians’ perspectives on the decision-making process about withholding/withdrawing LST in paediatric patients at EOL, specifically their perception of stakeholders, structure of the decision-making process about withholding/withdrawing LST, their ethical values, and other factors that can influence the decision-making.

### Involvement of the child in decision-making

Most physicians agreed about the importance of making decisions about withholding/withdrawing LST with parents. In only eight publications, physicians considered involving the child in the decision-making process; seven articles dealt with physicians and adolescent (10–19 years) patients [64].

#### Adolescents

Almost all physicians described in these publications agreed that adolescents should be involved in the decision-making process, because their self-awareness is forming, their values and beliefs are developing, and their cognitive capabilities are maturing. Even though adolescents cannot legally make the final decision about withholding/withdrawing LST, physicians apparently believed that adolescents are sufficiently autonomous to be at least involved in the decision-making. A statement from the American Academy of Pediatrics corroborated the notion that it is necessary to involve adolescents in the decision-making process, stating that ‘[e]ncouraging pediatric patients to actively explore options and to take on a greater role in their health care may promote empowerment and compliance with a treatment plan’ [65].

Our results are consistent with the results of two quantitative studies [66, 67], in which physicians agreed to involve adolescents in the decision-making process on withholding/withdrawing LST. Saudi Arabian physicians in the study of Alahmad et al. reported they considered children’s maturity more than chronological age to be important in deciding whether a child should be involved in the decision-making; however, in the end, they considered the age of 13–14 years to be an appropriate age to be involved in the decision-making [67]. In addition, most physicians in the study of Talati et al. agreed that adolescents should be included in the decision-making process especially when they agreed with their parents’ decision [66]. Thus, when the children and parents agreed with the decisions, 93 and 89% of physicians respected the treatment refusal of the children aged 16- and 11-year-old, respectively [66].

The conclusions of two published reviews [25, 68] corroborate our conclusions. In one review that suggested the strategy of integrating ethical justifications and guidelines, emotions, and communication skills while discussing disease progression with adolescents who were critically ill, physicians said it was a challenge to involve adolescents in the decision-making process because adolescents lack sufficient medical knowledge [68]. However, physicians also said that they should communicate more with adolescents to help them understand their poor prognosis and to help them develop an acceptable care plan.

In a narrative review of empirical studies on decision-making in cancer care treatments, adolescents were involved in the decisions, even though physicians were uncertain about how to involve them in an appropriate way [25]. Role-playing activities, organising art, storytelling, and poetry may help to promote adolescent participation [69, 70]. In general, physicians agreed to involve adolescents in the decision-making process, but they suggested that adolescents’ decision-making capacity should be assessed beforehand. To participate in decision-making, adolescents should be able to (1) comprehend medical information given by physicians; (2) rationally consider and make choices; (3) evaluate the benefits, risks, and hazards of decisions; and (4) possess a set of stable values to make decisions [71].

#### Young children

For younger children (1–10 years) [64], our analysis showed that there was no consensus among physicians on whether to involve them in the decision-making process. Our review revealed that many physicians were hesitant about involving young children in the decision-making process because they feared legal repercussions and because they have doubts about the children’s capacity for making reasoned decisions. Therefore, physicians considered more the viewpoints of parents than those of young children. For the same reasons, other quantitative studies showed that almost all physicians reported struggling with involving young children in the decision-making process when dealing with withholding/withdrawing LST; which is consistent with our results [66, 67, 72].

The Canadian Paediatric Society clarified how to appropriately involve younger children in the decision-making. They stated that patients’ capability for making decisions varied by age and clinical conditions, and this capability needs to be determined on a case-by-case basis [21]. Physicians should recognise and acknowledge young children’s dissent, respect and admit that children’s decision-making capacity is still developing, and provide them with appropriate information and options so that they know what to expect, e.g., by informing them about the treatment they would receive rather than asking for their consent [21, 65]. However, it is yet to be determined how to properly assess younger children’s capabilities and the extent to which they should be involved in the decision-making. Physicians in the study of Alahmad et al. indicated that they would use their extensive and long-term experience to assess young children’s capacity for making decisions, but no further details were provided [67]. Hence, how physicians evaluate young children’s decision-making capability in detail is still unclear and should be explored further.

### Involvement of parents in the decision-making process

Our analysis showed that almost all physicians agreed that parents should be involved in the decision-making process, not only because they are the appropriate surrogate decision-makers but also because children’s interests are intertwined with the interests of their parents. A statement from the Canadian Paediatric Society mentioned that ‘[m]ost preadolescent children need a substitute decision-maker to act on their behalf, and parents are usually the appropriate substitute decision-makers’ [21]. Our results are also supported by many other studies that also conclude that a family-centred and shared decision-making model of paediatric care is appropriate [21, 73–76]. This can be of value in maintaining a collaborative and long-term relationship with parents, even with disagreements. This key result in our review was in accordance with the findings in some quantitative studies. Physicians in the study of Randolph et al. considered family’s wishes as an extremely important factor helping them making EOL decisions about withholding/withdrawing LST [77]. 72% of physicians in the study of Bahus & Føerde [78]. and Aljethaily et al. [79] agreed that parents had the right to refuse or demand LST even though physicians considered the treatments were futile. In the study of Forbes et al. [80], 78.9% of senior physicians cited discussing withholding/withdrawing LST with parents as the most common facilitator which made them felt confident in their abilities. Half of the junior physicians also supported the experience of discussing withholding/withdrawing LST with parents as an important facilitator [80].

Our review also reveals that physicians face many difficulties when involving parents in the decision-making process for withholding/withdrawing LST. One is the conflict that can emerge between physicians and parents. For various reasons, physicians and parents may have different views about the process and the outcomes of the decision-making. This result from our review was consistent with the results of four quantitative studies that identified a barrier of discrepancies between physicians and parents [80–83]. In their studies, most paediatricians considered disagreements between physicians and parents about a child’s diagnosis and prognosis in the medical assessment to be an important issue in the decision-making process [80–83]. Sometimes, physicians did not know how to respond if the parents’ requests or demands were not in what they perceived to be in the child’s best interest. For instance, paediatricians considered that parents’ request of continuing LST did not match the child’s best interest, but they were not sure how to solve the conflict because they have little knowledge about assisting parents to weigh up the pros and cons of various treatment options [80].

The American Academy of Pediatrics [20] and the Canadian Paediatric Society [21] suggest three ways to deal with conflicts between physicians and parents. First, they encourage that all stakeholders embrace open communication, which is always essential for conflict resolution. Second, if disagreement persists, physicians are advised to postpone the proposed interventions, if possible. Third, it is imperative to recruit ethics support, including ethics consultation and moral case deliberation. Ethics support is considered to be an effective way to resolve conflicts through clinical supervision and face-to-face discussions, and by guiding physicians and parents to clarify the care goals, maintaining the best interests of the child, and making decisions jointly [11, 20, 84–89]. This supports and enhances interprofessional well-being in healthcare team interactions and interactions with parents, helps to comfort HCPs while dealing with ethical challenges and improves the care quality for children and families [89].

### Lack of physicians’ training and education in paediatric palliative care

A prominent observation emerging from our review was physicians’ lack of PPC education and training. This deficit has been described as one of the main barriers of the decision-making process when dealing with withholding/withdrawing LST in paediatric patients at EOL. Our observations corroborate those of other empirical studies. Many paediatricians reported that they did not receive adequate PPC training [90] or ACP training [91–93]. This lack of adequate training distresses paediatricians, because they are inexperienced and unsure about communicating with parents about the child’s EOL issues, initiating PPC, or providing pain management at EOL [94–97]. Paediatricians expressed a desire to receive PPC training so that they could more effectively provide PPC [97]. PPC training bolstered paediatricians’ confidence when they had to provide PPC to dying children and their families, mostly because they had mastered relevant knowledge and skills [94]. Finally, institutional support for PPC training was identified as an important facilitator of PPC in paediatrics [98, 99].

### Strengths and limitations

The main strengths of this review lie in the rigorous and systematic approach adopted. We employed systematically developed search strings, clear inclusion and exclusion criteria for publication selection, and a well-tested and comprehensive data extraction and synthesis process. The inclusion process and quality appraisal of the 30 included articles were conducted by two of the authors in an independent way. Following the qualitative analysis guide of QUAGOL [32, 33], we continuously reflected on the data in a critical and conceptual way. Another strength is that the included publications originated from studies conducted in 16 different countries, including those in North America, Europe, Oceania, and Asia. Thus, our observations and conclusions are based on data obtained from diverse physicians across the globe, and from different cultures and legislative systems, providing an international perspective. Although we did not constrain our literature search to a truncated period, all the publications that met our inclusion criteria were published after the year 2000, ensuring a sampling of the contemporary views of current physicians.

Nevertheless, our review had some limitations. Firstly, our literature search limited the publication language of articles to English. English is the only common language that the four authors can understand and use. In addition, we extracted a large amount of relevant information from the included 30 English articles, and we considered we got enough data for analysis. Therefore, we included publications in English only. However, because of the exclusive focus on articles in English, the generalisability of our conclusions may be somewhat limited. Future similar reviews that also include foreign-language publications will determine whether our conclusions need tempering. Secondly, despite strict inclusion/exclusion criteria, studies reporting on self-reported perspectives of physicians might be affected by social desirability bias. Thirdly, most of the included publications reported on studies that were conducted in high-income countries, which might have introduced cultural bias to our results. Specifically, as only a few studies were done in middle-income countries, religious and cultural differences may be masked somewhat. Studies conducted in low-income countries were not presented in this review.

## Conclusions

This review synthesised physicians’ perceptions found in the literature about withholding/withdrawing LST in paediatrics. We determined from our analysis of the literature that physicians generally agreed to share the decision-making with parents and the child. Our synthesis of physicians’ perceptions supports a view that the decision-making process is generally divided into three stages: (1) early preparation of considering a decision through ACP; (2) information giving and receiving; and (3) arriving at the final decision about withholding/withdrawing LST. Most physicians felt that their main work was related to advocating for the best interest of the child. Additionally, moderating factors related to physicians’ and parents’ views were identified. Finally, approaches for evaluating young children’s capacity for making EOL decisions remain unclear and needs to be explored further. In conclusion, this review can help HCPs and other stakeholders to better understand how and why physicians’ decide on withholding/withdrawing LST at EOL in paediatric patients; some conclusions may be relevant for other vulnerable patient populations at EOL.

## Supplementary Information

**Additional file 1: Supplementary Material 1.** Overview of bibliographic databases searched, search strings used, and search results of articles identified.

## Data Availability

All data generated or analysed during this study are included in this published article and its supplementary information files.

## Abbreviations

EOL: End-of-life
PPC: Paediatric palliative care
LST: Life-sustaining treatments
PICUs: Paediatric intensive care units
CASP: Critical Skills Appraisal Program
QUAGOL: Qualitative Analysis Guide of Leuven
ACP: Advance care planning
HCPs: Healthcare providers
